# Supplementary material for: Photooxidation triggered ultralong afterglow in carbon nanodots
Source: Nat Commun. 2024 Mar 15;15:2365. doi: 10.1038/s41467-024-46668-z (PMC10943204; doi:10.1038/s41467-024-46668-z)
Supplement: Supplementary file 1 — Supplementary Information [file 41467_2024_46668_MOESM1_ESM.pdf]

Supplementary Information

Photooxidation triggered ultralong afterglow in carbon nanodots

Guang-Song Zheng[1,#], Cheng-Long Shen[1,#], Chun-Yao Niu[1,#], Qing Lou*[1], Tian-Ci Jiang[2,3], Peng-Fei Li[2,3], Xiao-Jing Shi[4], Run-Wei Song[1], Yuan Deng[1], Chao-Fan Lv[1], Kai-Kai Liu[1], Jin-Hao Zang[1], Zhe Cheng[2,3], Lin Dong[1], Chong-Xin Shan*[1]

[1]Henan Key Laboratory of Diamond Optoelectronic Materials and Devices, Key Laboratory of Material Physics, Ministry of Education, and School of Physics and Microelectronics, Zhengzhou University, Zhengzhou 450052, China

[2]Department of Respiratory and Critical Care Medicine, The First Affiliated Hospital of Zhengzhou University, Zhengzhou, China

[3]Henan Key Laboratory for Pharmacology of Liver Diseases, Institute of Medical and Pharmaceutical Sciences, Zhengzhou University, Zhengzhou, China

[4]Academy of Medical Sciences, Zhengzhou University, Zhengzhou, China

[#]These authors contributed equally: Guang-Song Zheng, Cheng-Long Shen, Chun-Yao Niu.

E-mail: louqing1986@zzu.edu.cn (Q. Lou), cxshan@zzu.edu.cn (C. X. Shan)

This file includes:

Supplementary Note 1 to 15

Supplementary Fig. 1 to 49

Supplementary Table 1 and 2

Supplementary Note 1

Production yields: As illustrated in Supplementary Fig. 1, the CDs-1 from 1.0006 g plant source and obtained 0.0135 g CDs, thus the production yields of CDs is about 1.35%.

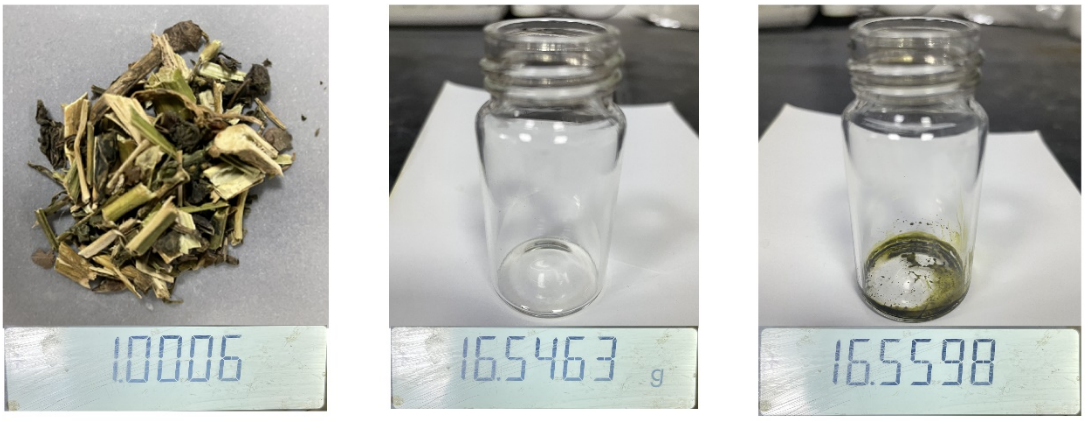

Supplementary Fig. 1. Mass of the source, initial flask and the flask with the products.

Supplementary Note 2

A potential strategy to improve the production yield: Generally, industrial production often involves using larger high-pressure vessels to expand the reaction and yield. Thus, we have compared the production yield of CDs using the poly (tetrafluoroethylene) (Teflon)-lined autoclaves with different volumes. As shown in the Supplementary Fig. 2a, 20, 50 and 100 mL autoclaves were used to prepare the CDs with the same source of 1 g plants and 10, 20 and 50 mL ethanol, respectively. As a result, the weights of the obtained CDs finally were 0.009, 0.0133 and 0.0287 g, and the corresponding production yields were 0.9%, 1.3% and 2.9%, respectively (Supplementary Fig. 2b to 2d). The results clearly indicate that large reaction chamber can effectively improve the production yields of CDs, which may be attributed to the solvent to raw material contact ratio and the shift of reaction balance. We believe the potential strategy to improve the production yield of CDs by enlarging the reaction chamber is also suitable for the large-scale industrial production.

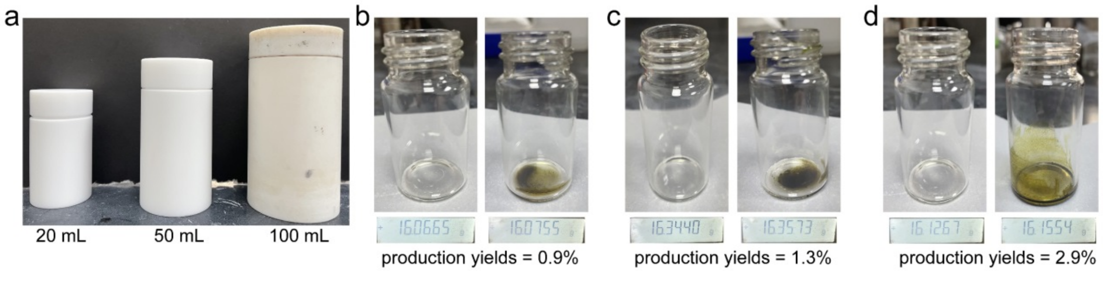

Supplementary Fig. 2. a The photograph of the poly (tetrafluoroethylene) (Teflon)-lined autoclaves

with different volumes. **b** Weights of the initial flask and the flask with the CD products from the 25 mL reactors. **c** Weights of the initial flask and the flask with the CD products from the 50 mL reactors. **d** Weights of the initial flask and the flask with the CD products from the 100 mL reactors.

Supplementary Note 3

The replicability of CDs: The Solanum nigrum L used for the preparation of CDs was purchased from Bozhou Kangyiyin Biotechnology Co., Ltd., without undergoing any additional treatment. The mixture solutions were transferred to a poly (tetrafluoroethylene) Teflon-lined autoclave (50 mL) and heated for 4 h at 80°C, 120°C, and 160°C, respectively. After the products cooled down to room temperature, the solutions were filtered through a 0.22 μm polyether sulfone membrane to remove large particles, and the obtained crude products were further purified via silica column chromatography using a mixture of ethyl acetate and petroleum ether as the eluent. After collecting CDs by removing the solvent under reduced pressure, CDs-1， CDs-2 and CDs-3 were obtained for further characterization.

Secondly, we have prepared three batches to investigate the variations of the properties of the CDs. As illustrated in Supplementary Fig. 3 and Fig. 4, these batches of CDs were tested with fluorescence spectra and absorption spectra and it can be observed that these CDs exhibited the same emission wavelengths and UV-vis absorption. Meanwhile, the PLQY of each batch were measured, and the results revealed that all CDs possess nearly PLQY (Supplementary Fig. 5), indicating the excellent repeatability of the approach to the CDs from batch to batch.

The replicability of CDs prepared with the plants purchased from different supplier: The plant sources (*Solanum nigrum L*) for the production of carbon dots (CDs) were purchased from different suppliers, including Bozhou Kangyiyin Biotechnology Co., Ltd., Henan Green He Pharmaceutical Co., Ltd., and Bozhou Haoyitang Biotechnology Co., Ltd. The afterglow CDs were prepared with the plant sources from the three suppliers under the same condition of solvothermal and chromatographic treatment (Supplementary Fig. 6a, 6f and 6k). The potential variations of these CDs were investigated. As illustrated in Supplementary Fig. 6b, 6c, 6g, 6h, 6l, and 6m, these batches of CDs from different suppliers were checked with fluorescence and absorption spectra. And the results revealed that these CDs possessed almost the same fluorescence and UV-vis absorption

spectra without obvious peak shift. Meanwhile, the PL QYs of these batches of CDs also demonstrated the similar PL QYs of 28.42%, 27.86% and 28.44% for these CDs (Supplementary Fig. 6d, 6i and 6n), indicating the excellent repeatability of the preparation approach to the CDs from batch to batch. In addition, the production yields of CDs prepared from different suppliers were also investigated. As shown in the Supplementary Fig. 6e, 6j and 6o, the production yields of 1.3%, 0.8% and 1.8% could be obtained from different suppliers. The above results clearly indicated that the production of CDs is reproducible if a different batch from a different supplier is used while only there is difference in production yields.

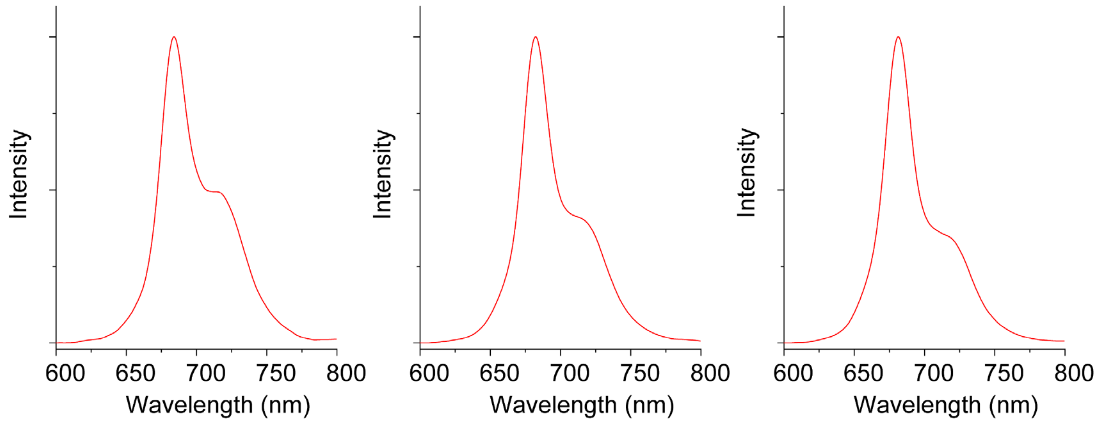

Supplementary Fig. 3. Fluorescence spectra of the CDs-1 prepared from three batches.

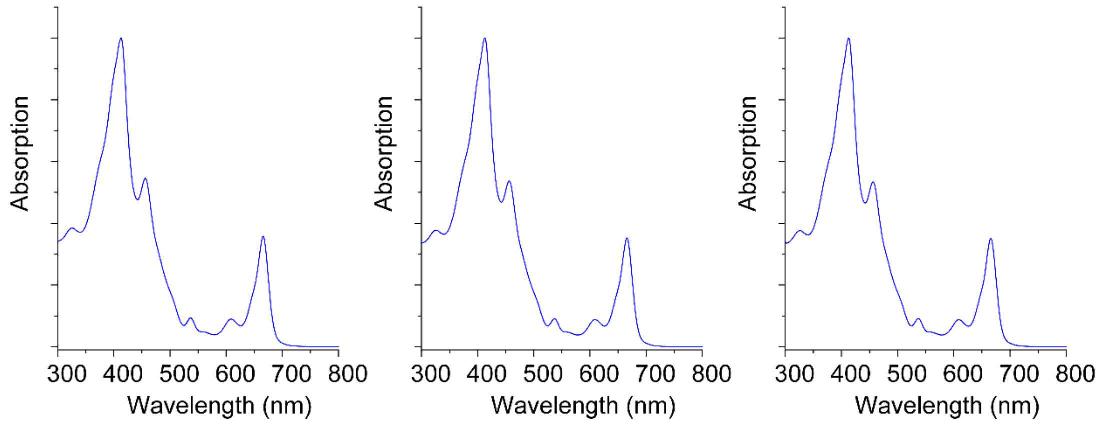

Supplementary Fig. 4. UV-vis spectra of the CDs-1 prepared from three batches.

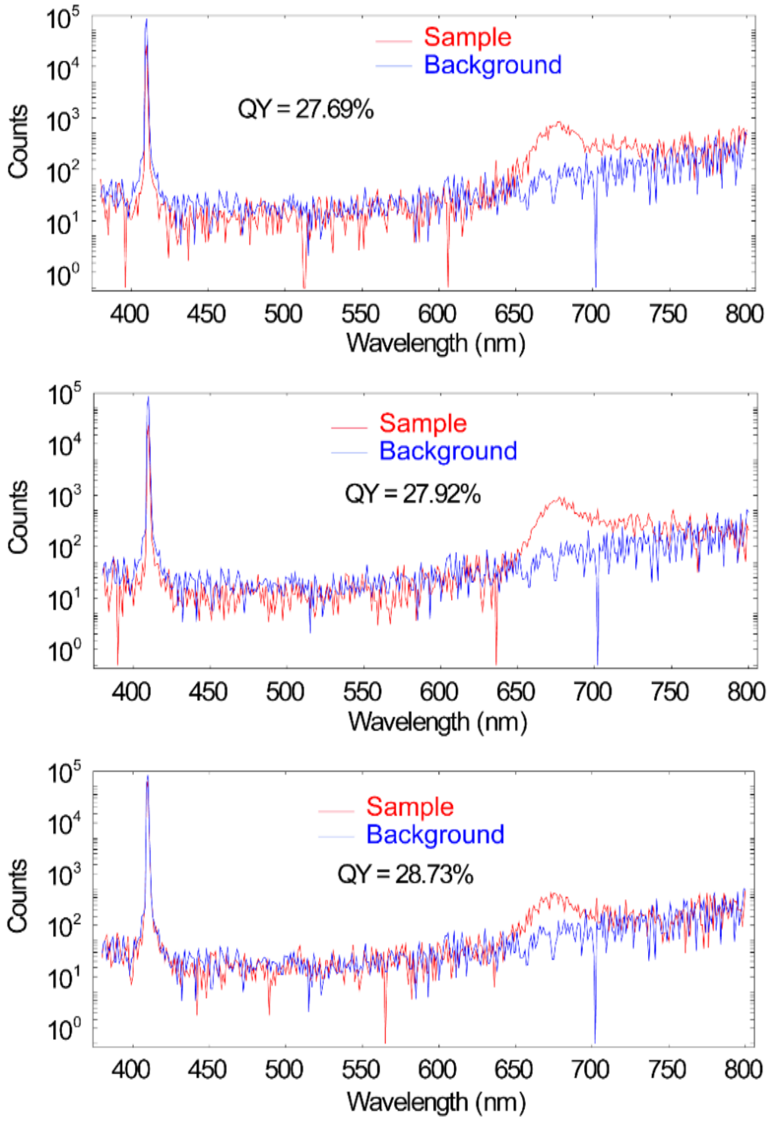

Supplementary Fig. 5. PLQY of the CDs-1 prepared from three batches.

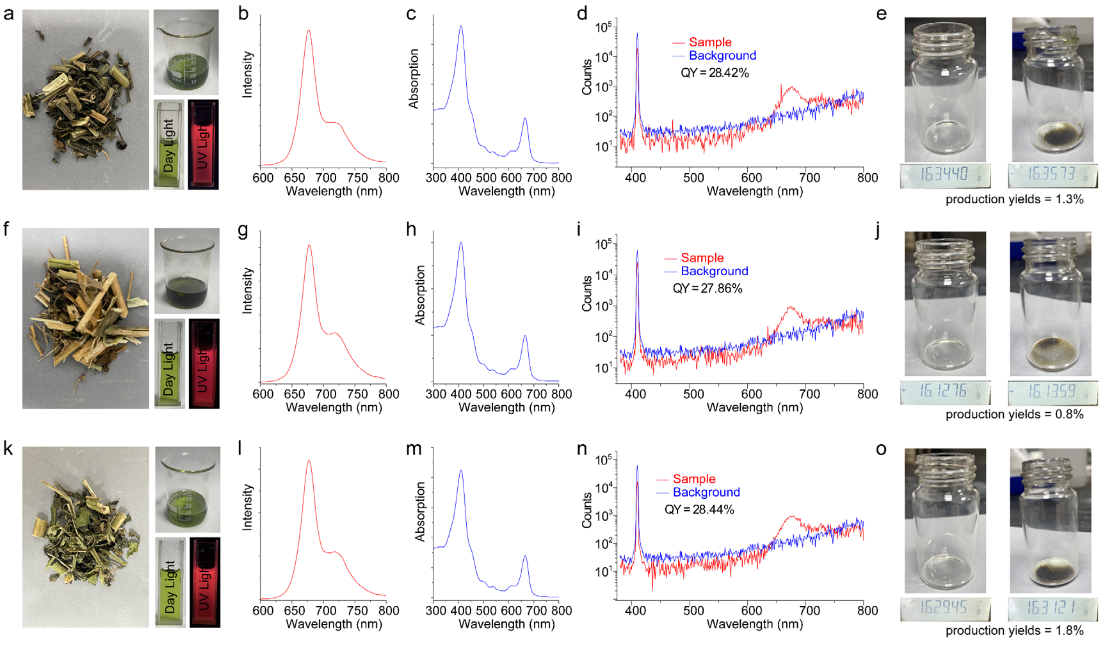

Supplementary Fig. 6. a-e a The photograph of Solanum nigrum L and **b** fluorescence spectrum, **c** absorption spectrum, **d** PLQY and **e** production yield of the CDs prepared from the source purchased from Bozhou Kangyiyin Biotechnology Co., Ltd. **f-j f** The photograph of Solanum nigrum L and **g** fluorescence spectrum, **h** absorption spectrum, **i** PLQY and **j** production yield of the CDs prepared from the source purchased from Henan Green He Pharmaceutical Co., Ltd. **k-o k** The photograph of Solanum nigrum L and **l** fluorescence spectrum, **m** absorption spectrum, **n** PL QY and **o** production yield of the CDs prepared from the source purchased from Bozhou Haoyitang Biotechnology Co., Ltd.

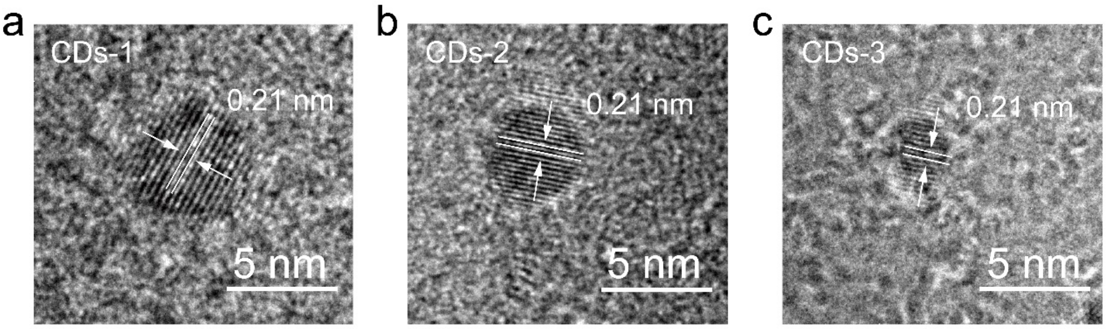

Supplementary Fig. 7. The high-resolution TEM images of CDs-1, CDs-2, and CDs-3. The imaging in (**a, b, c**) derived from three independent measurements.

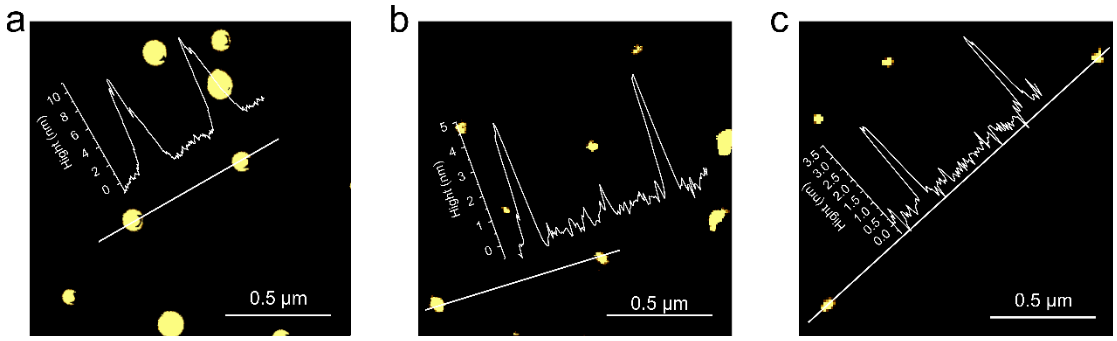

Supplementary Fig. 8. The AFM images of CDs-1, CDs-2, and CDs-3.

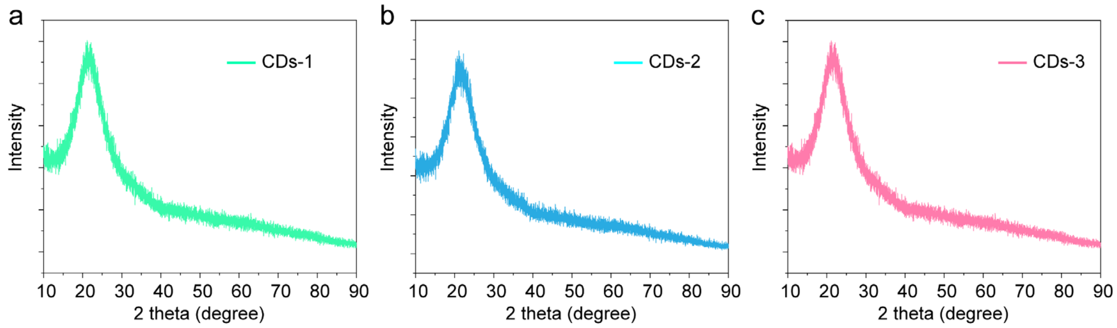

Supplementary Fig. 9. The XRD pattern of CDs-1, CDs-2 and CDs-3.

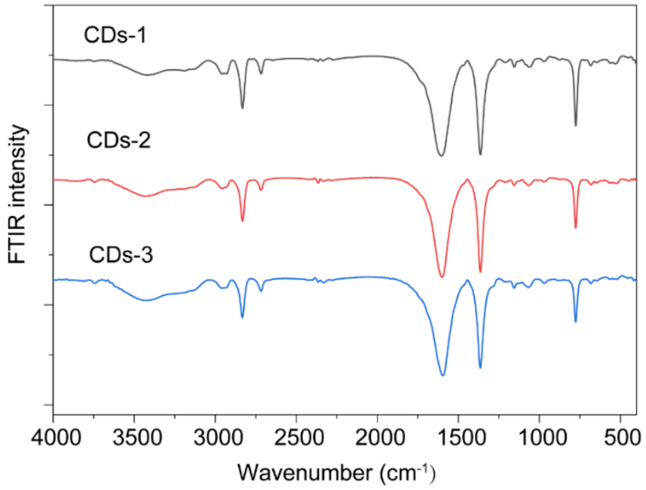

Supplementary Fig. 10. FT-IR spectra of these CDs-1, CDs-2 and CDs-3.

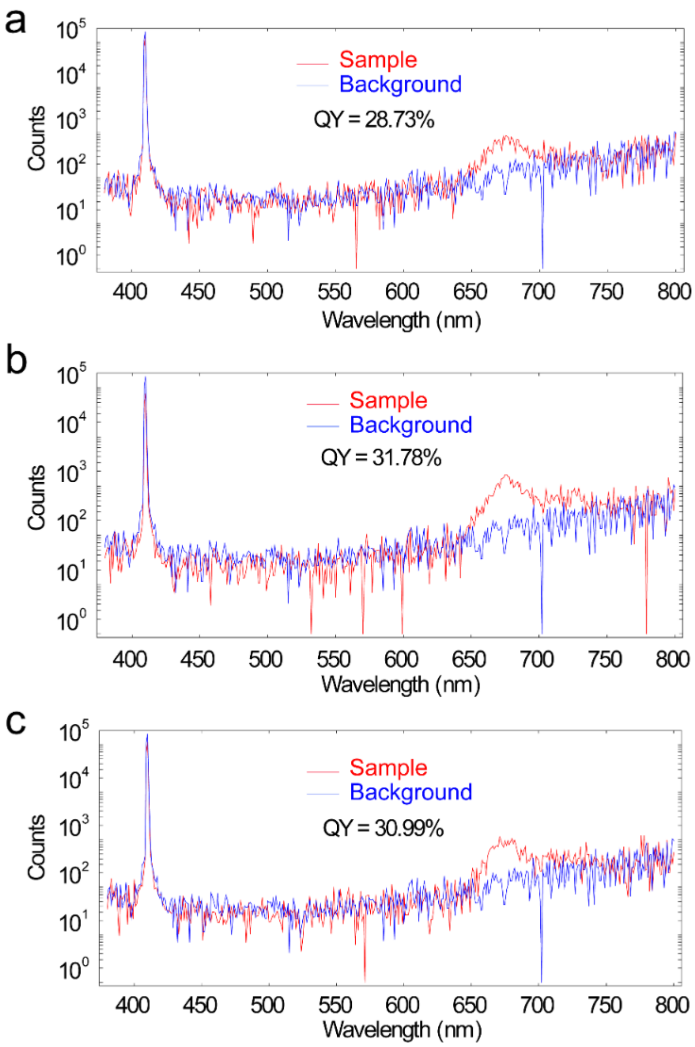

Supplementary Fig. 11. PL spectra with and without sample, and the PL QY of **a** CDs-1, **b** CDs-2 and **c** CDs-3.

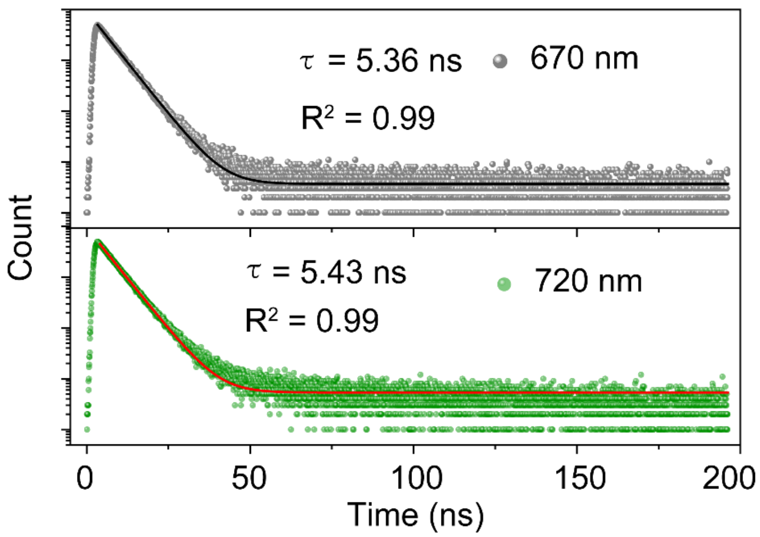

Supplementary Fig. 12. Time resolved decay spectra of the CDs with 670 nm and 720 nm.

Supplementary Note 4

The fluorescence mechanism of CDs: To explore the fluorescence mechanism of CDs, the excitation–emission matrix of the CDs was tested. As shown in the Supplementary Fig. 13a, the CDs present a stable luminescence center with independent excitation wavelength. For the emission of CDs at 670 nm, the absorption and emission spectra of CDs show an ultra-small Stokes shift of 12 nm (Supplementary Fig. 13b), indicating the interbond exciton recombination and weak electron-phonon coupling interactions. This implies that the emission at 670 nm may originate from the band-edge recombination of CDs.

To further evaluate the intrinsic properties of photo-excitons in CDs, we have plotted a two-dimensional false-color diagram of the temperature-dependent PL spectrum (Supplementary Fig. 14a). As the temperature increases from 80 to 300 K, the integral PL intensity at 670 nm of CDs decreases to 23%, which indicates high thermal stability for the radiative recombination of the CDs. The corresponding exciton binding energy can be extracted by plotting the integral area of PL emission intensity as a function of temperature according to the following equation:

$$I(\mathrm{T}) = \frac{I_0}{1 + \mathrm{A}\exp(-E_\mathrm{b} / k_\mathrm{B}T)} \quad (1)$$

Where I_0 represents the integral intensity of PL emission at 0 K, A is the proportionality constant, E_b is the exciton binding energy, and k_B is the Boltzmann constant. As shown in Supplementary Fig. 14b, the fitting analysis results show that the CDs have a relatively large E_b of 47.34 meV, indicating their strong exciton recombination ability. High E_b and fast emissivity indicate that the CDs has extraordinary luminescence properties. The emission bandwidth of CDs is considered to be closely related to the strength of electron-phonon coupling. To determine this strength, the Huang-Rhys factor (S) is introduced as a metric to estimate the coupling strength by plotting full width at half maximum (FWHM) as a function of the inverse temperature and fitting with the following equation:

$$\mathrm{FWHM}(\Gamma) = 2.36\sqrt{S}\hbar\omega_{\mathrm{photon}}\sqrt{\coth\frac{\hbar\omega_{\mathrm{photon}}}{2k_\mathrm{B}T}} \quad (2)$$

Where S is the yellow-Rhys factor, ω refers to the frequency of phonons, S is estimated to be 0.13 (Supplementary Fig. 14c). The small S value confirms the weak coupling between electron transitions and lattice phonons in the CDs. In addition, the PL position remains relatively unchanged

in the temperature ranging from 80 to 300 K, indicating that the electron-phonon interaction is relatively weak. Based on these results, it can be inferred that the CDs exhibit high structural stiffness and weak electron-phonon interactions, which may contribute to the stable red emission.[1]

For the emission at 720 nm of CDs, there is an approximately 60 nm Stokes shift and a larger FWHM. Additionally, the fluorescence lifetime at 720 nm is measured at 5.43 ns (Supplementary Fig. 12), indicating a faster radiative recombination. Moreover, the fluorescence lifetimes at 720 and 670 nm (5.36 ns) exhibit certain differences, suggesting distinct emission sources. According to previous reports,[2-4] it may originate from the surface-localized excitonic vibrational fine emission bands of CDs. This hypothesis can also be confirmed by the spectral changes of the CDs after the surface passivation of F127. As shown in the Supplementary Fig. 15a, compared with the pure CDs, the surface-passivated p-CDs present a relatively weaken fluorescence emission at 720 nm owing to that the surface passivation of F127 on CDs limits the vibration emission on the surface. Hence, 720 nm related emission is corresponding to the surface-localized excitonic vibrational fine emission bands in the CDs, which is also reflected in the apparent discrepancy presented in the fluorescence and photooxidation afterglow spectra of the CDs (Supplementary Fig. 15b).

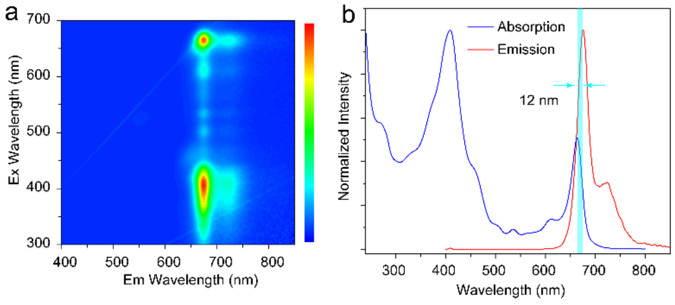

Supplementary Fig. 13. a The excitation–emission matrix of the CDs solution. **b** The UV-Vis absorption and corresponding PL spectrum of CDs.

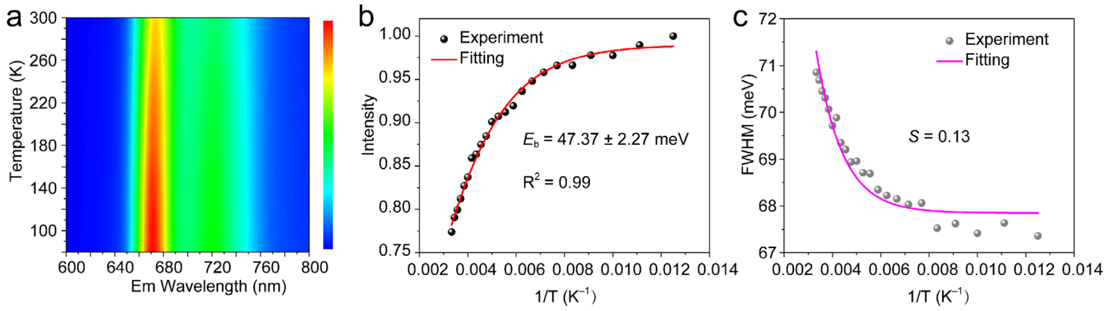

Supplementary Fig. 14. a The PL emission of the CDs in the temperature range from 80 to 300 K.

b Integrated PL intensity and **c** FWHM as a function of reciprocal temperature.

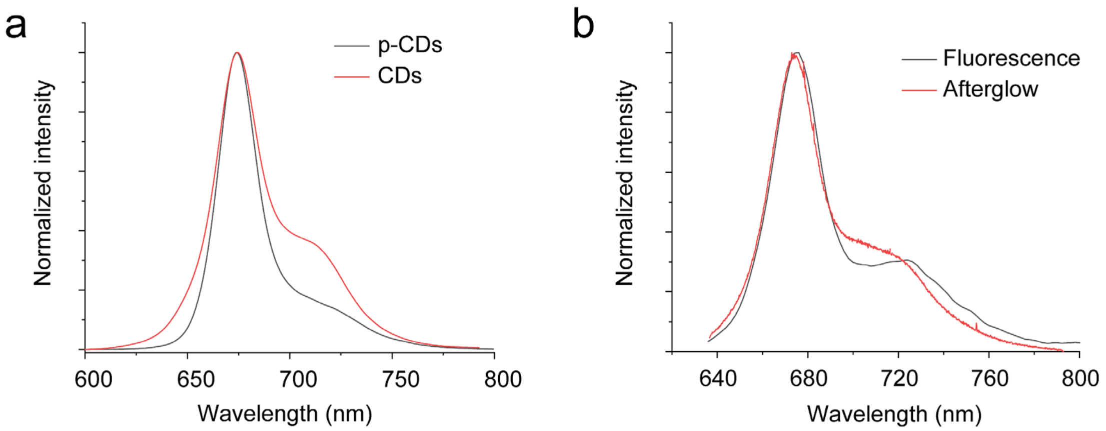

Supplementary Fig. 15. a Fluorescence spectrum of p-CDs and CDs. b The Fluorescence and afterglow spectra of CDs.

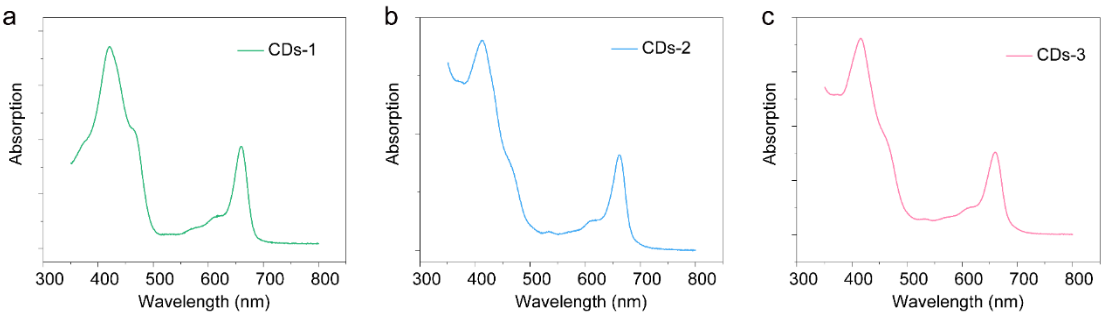

Supplementary Fig. 16. The absorption spectra of CDs-1, CDs-2 and CDs-3.

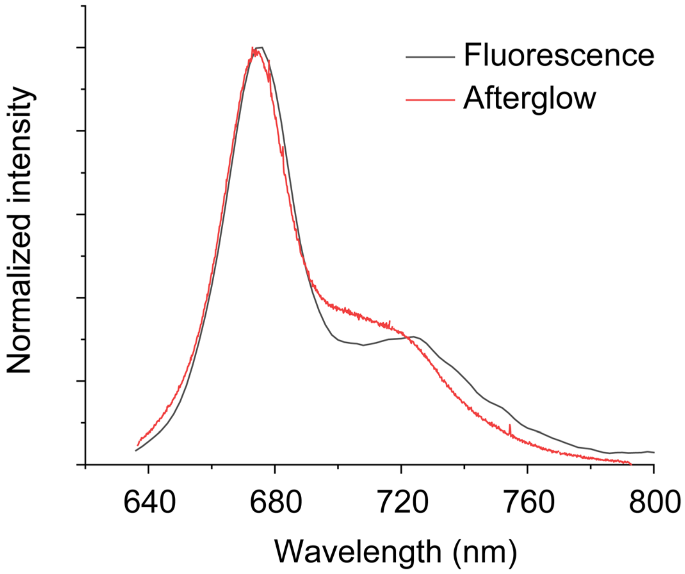

Supplementary Fig. 17. The Fluorescence and afterglow spectra of CDs.

Supplementary Note 5

The repeatability of the afterglow lifetime of CDs: We have conducted the repeated tests on the lifetimes of CDs-1, CDs-2, and CDs-3. As depicted in Supplementary Fig. 18, Fig. 19 and Fig. 20, the afterglow decay exhibit almost the same performance, indicating the excellent repeatability of lifetimes for the CDs-1, CDs-2, and CDs-3 (Supplementary Fig. 21).

Experimental Method: The CDs ethanol solution (2 mL, 1 mg mL^{-1}) was injected into a 5 mL PVC tube. The solution was exposed with 660 nm laser (2 min, 1.5 W cm^{-2}). Subsequently, 200 µL of the illuminated CDs solution was transferred to a 96-well black enzyme-linked immunosorbent assay (ELISA) plate. Afterglow testing was conducted using a Bioluminescence mode in an in vivo imaging system with a 670 nm filter, 2 min post-illumination, and 5 min intervals for 4 h.

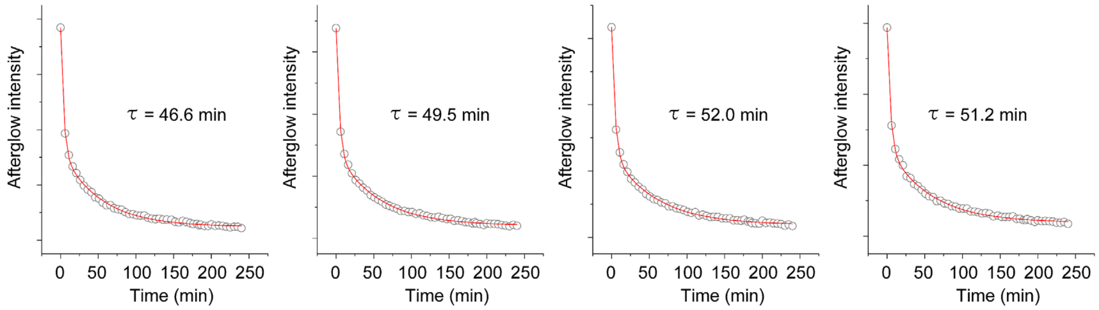

Supplementary Fig. 18. Afterglow lifetime curves of the CDs-1.

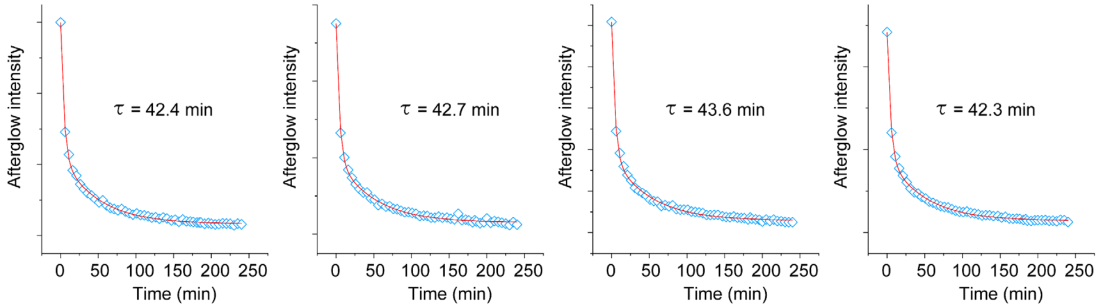

Supplementary Fig. 19. Afterglow lifetime curves of the CDs-2.

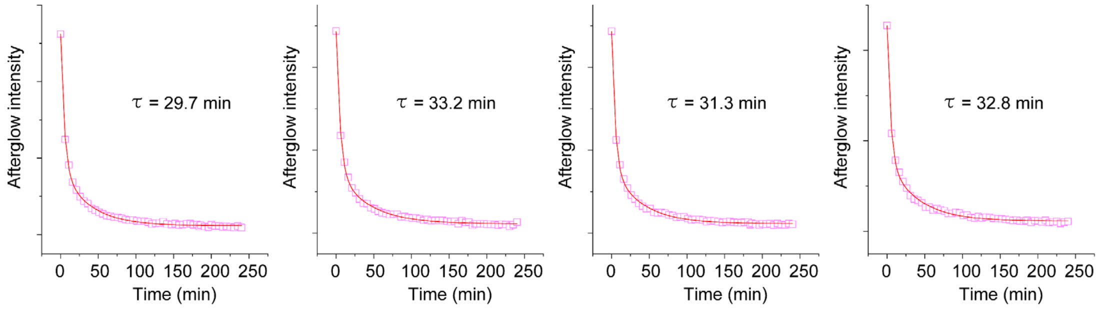

Supplementary Fig. 20. Afterglow lifetime curves of the CDs-3.

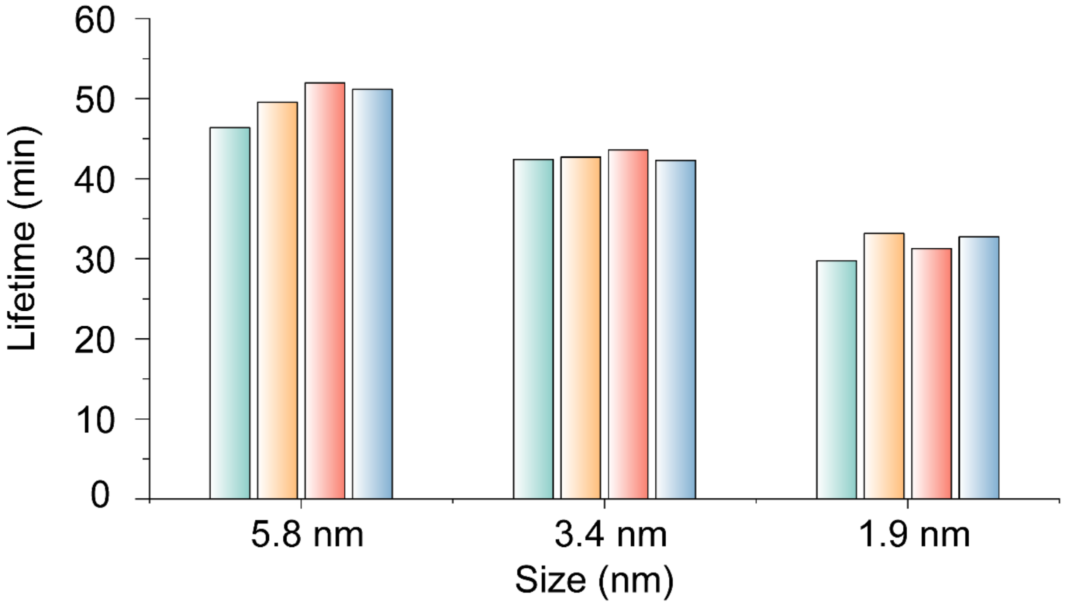

Supplementary Fig. 21. The lifetime of CD-1、CD-2 and CD-3.

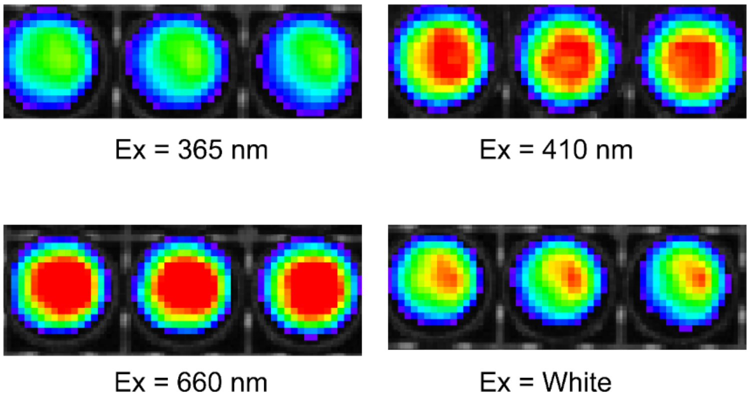

Supplementary Fig. 22. The pre-irradiation wavelength of afterglow from UV to deep-red light region and include the white light.

Supplementary Note 6

As reported in previous literatures,[3-5] the fluorescence emission of CDs is related to the doping of N atoms. Generally, the pyridine N and pyrrole N can contribute to the formation of a π-conjugated system with a pair of p-electrons in the CDs, providing a strong radiative recombination center. To investigate the potential evolution of the CDs under light irradiation, we have compared the N species of the CDs before and after lasting irradiation. As shown in the Supplementary Fig. S23 and S24, the N 1s spectra exhibit three peaks assigned to pyridinic N, pyrrolic N, and graphitic N for the CDs before and after light irradiation. And the corresponding content and type of N species did

not change significantly, indicating that photooxidation primarily occurred on the surface of CDs. As a result, the non-N-related non-radiative recombination centers like –COOH can be generated,[6-8] leading to the slight reduction in fluorescence and afterglow intensity of CDs after the photooxidation process.

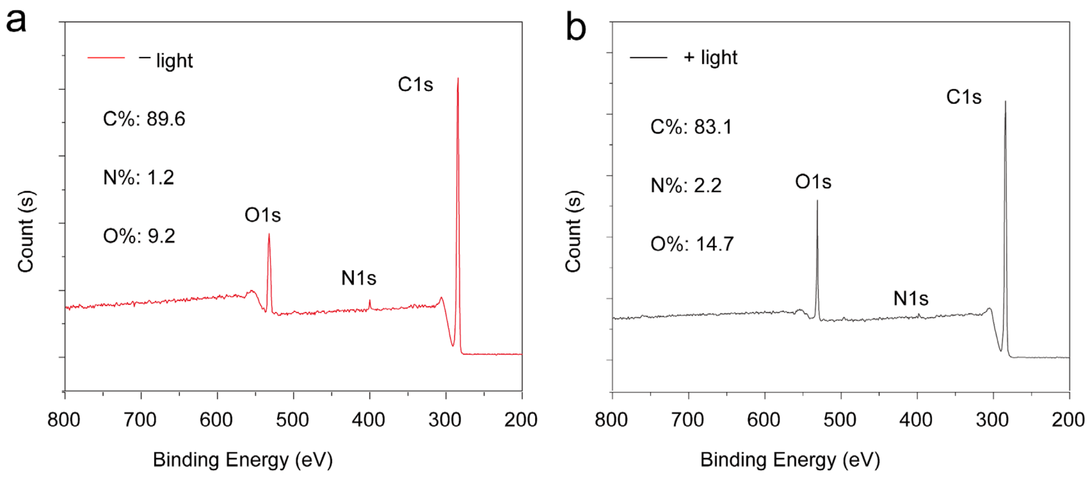

Supplementary Fig. 23. The full survey XPS of the CDs-1 before and after light irradiation.

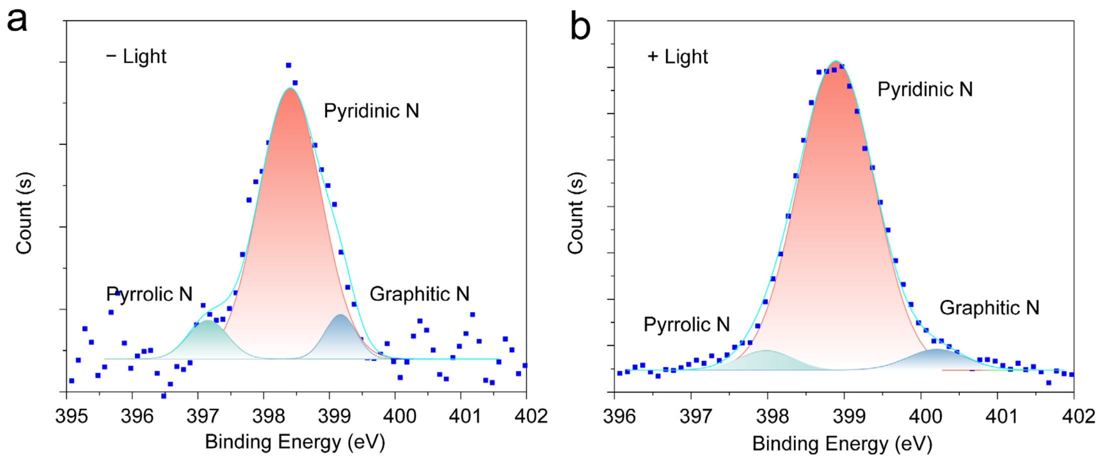

Supplementary Fig. 24. a The high-resolution XPS N1s spectrum of the CDs before light irradiation. **b** The high-resolution XPS N1s spectrum of the CDs after light irradiation.

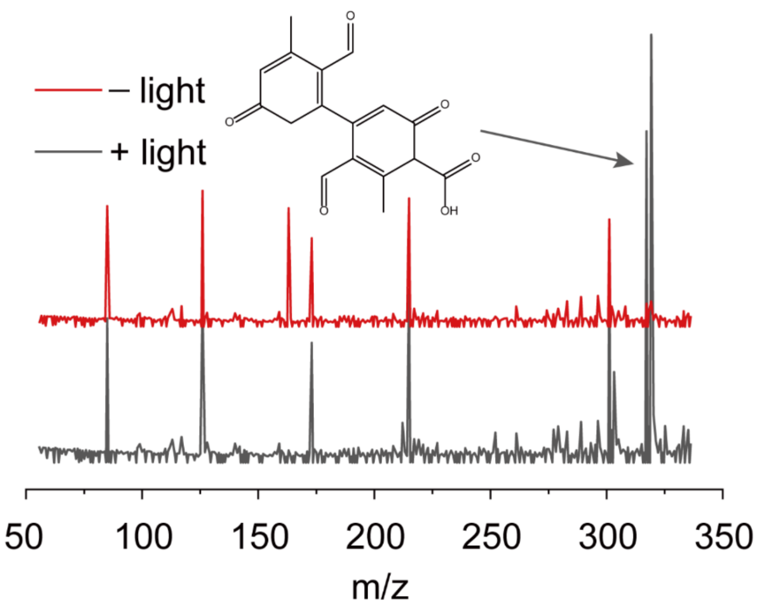

Supplementary Fig. 25. The MS spectrum of CDs before and after light irradiation treatment.

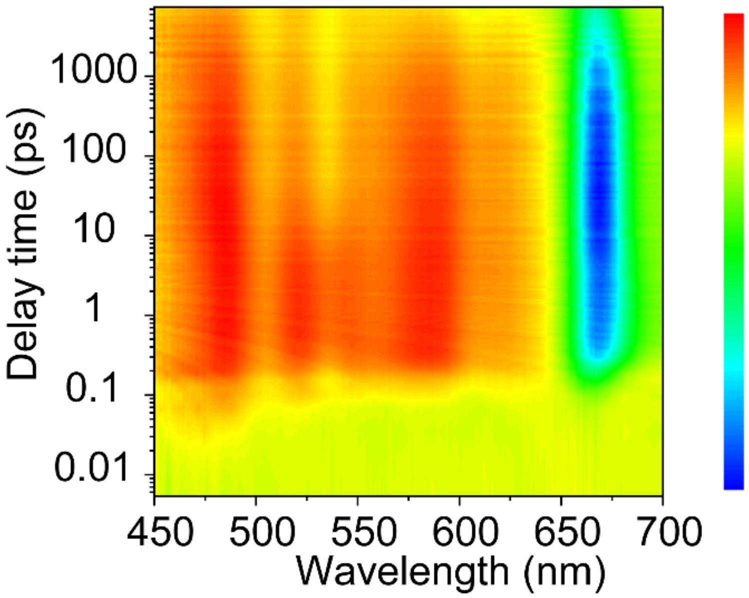

Supplementary Fig. 26. 3D pseudocolor map of transient absorption (TA) spectra of CDs with the probe wavelength excited at 410 nm.

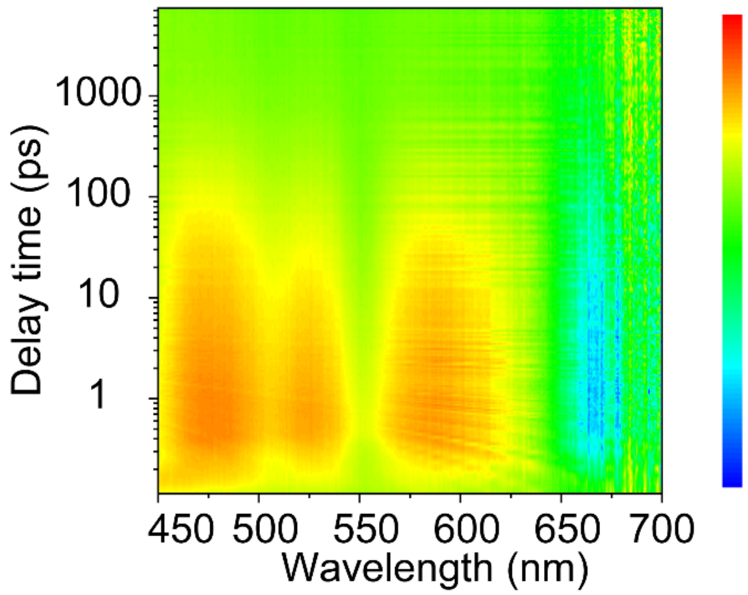

Supplementary Fig. 27. 3D pseudocolor map of transient absorption (TA) spectra of CDs with the probe wavelength excited at 410 nm after light irradiation.

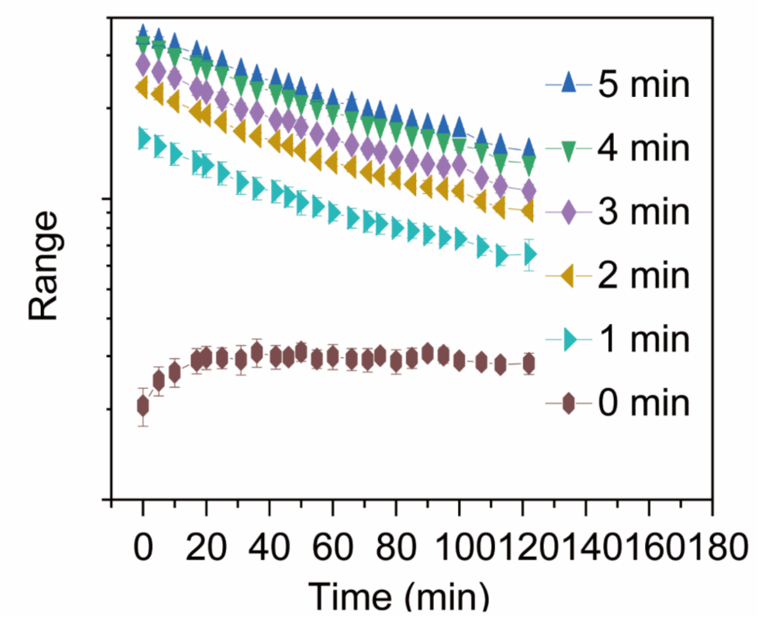

Supplementary Fig. 28. Decay of afterglow signal of CDs (1 mg mL^{-1}) over time at room temperature after light irradiation (660 nm, 1.5 W cm^{-2}, from 0 to 5 min).

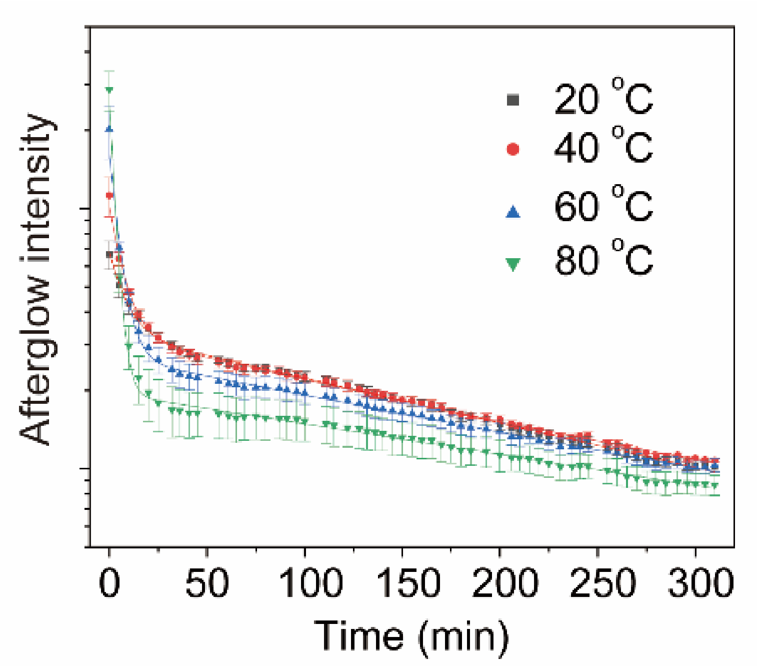

Supplementary Fig. 29. Decay of afterglow signal of CDs (1 mg mL^{-1}) over time at 20, 40, 60 and 80°C after light irradiation (660 nm, 1.5 W cm^{-2}, 2 min).

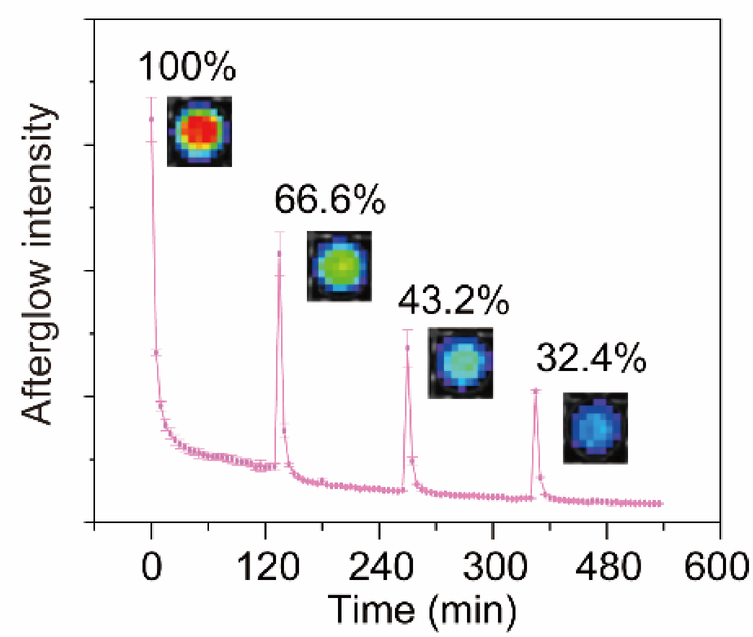

Supplementary Fig. 30. Decay of afterglow signal of CDs (1mg mL^{-1}) with time at room temperature after light irradiation (660nm, 1.5 W cm^{-2}, 30 min), repeated four times.

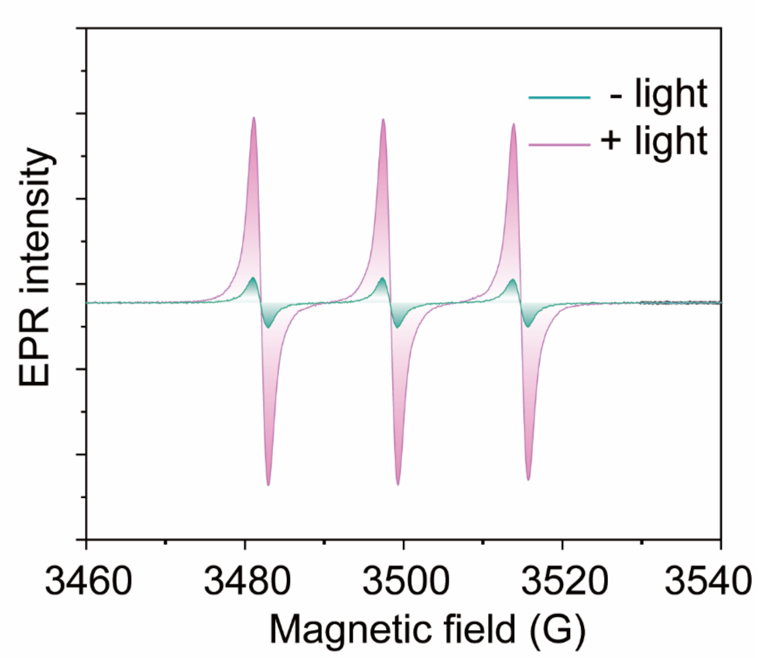

Supplementary Fig. 31. EPR spectrum of CDs before and after light irradiation.

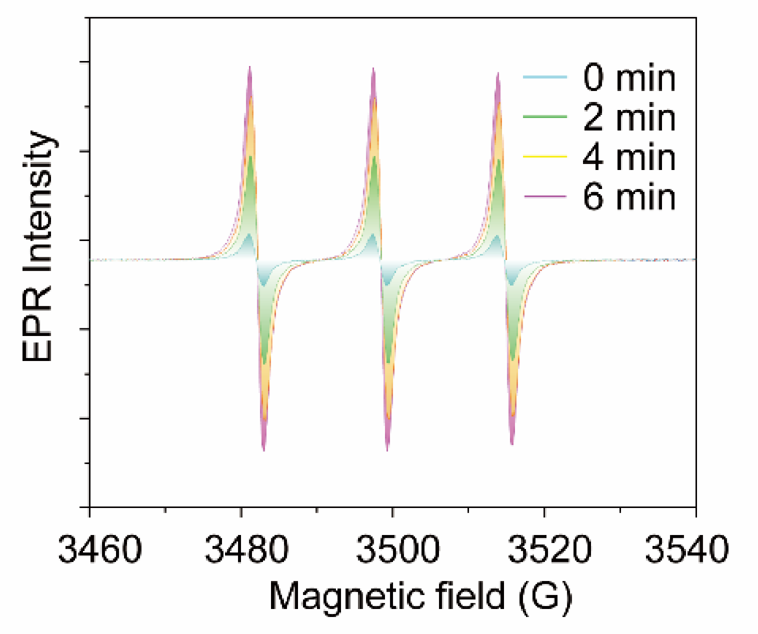

Supplementary Fig. 32. EPR spectrum of CDs with the light irradiation at room temperature (660 nm, 1.5 W cm^{-2}, from 0 to 5 min).

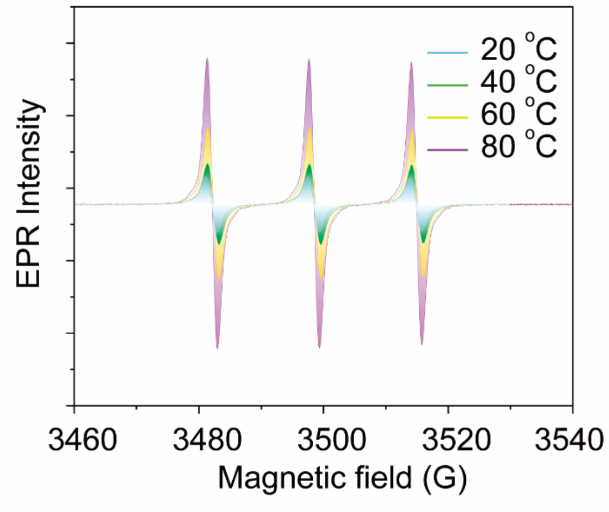

Supplementary Fig. 33. EPR spectrum of CDs (1 mg/mL) over time at 20, 40, 60 and 80°C after light irradiation (660 nm, 1.5 W cm^{-2}, 2 min).

Supplementary Note 7

The possible photooxidative afterglow of the solid-state CDs has been tested. As depicted in the Supplementary Fig. 34, the CDs in the solid-state exhibit prominent afterglow emission when they are exposed to air. However, when the solid-state CDs are encapsulated in epoxy resin to isolate oxygen, the CDs present no afterglow emission after irradiation (Supplementary Fig. 35). This result clearly proves that the participation of oxygen is the necessary requirement for the afterglow emission of CDs. Different from phosphorescence or thermally activated delayed fluorescence, the photooxidation afterglow of CDs originates from the continuous oxidation of CDs by singlet oxygen, where oxygen is active involvement in the photooxidation process.

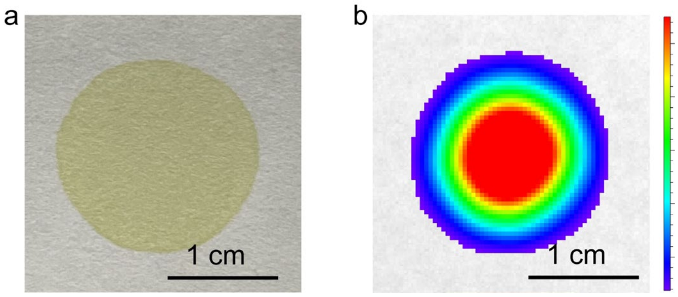

Supplementary Fig. 34. The photograph of the CDs in solid exposed to air in sunlight. The afterglow of the CDs in solid exposed to air with the irradiation (660 nm, 1.5 W cm^{-2}, 2 min).

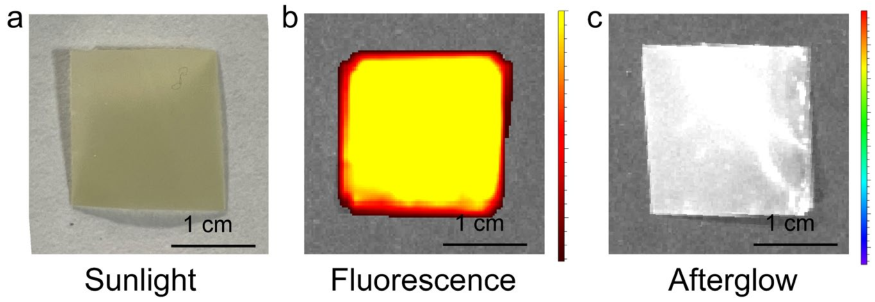

Supplementary Fig. 35. The photograph of the CDs in solid is encapsulated in epoxy resin in sunlight. The afterglow of the CDs in solid is encapsulated in epoxy resin with the irradiation (660 nm, 1.5 W cm^{-2}, 2 min).

Supplementary Note 8

The afterglow performances of the CDs in common oxidants (active nitrogen, $ONOO^-$; hypochlorous acid, ClO^-; hydrogen peroxide, H_2O_2). As illustrated in Supplementary Fig. 36, the $ONOO^-$, ClO^-, and H_2O_2 can trigger the photooxidation afterglow of the CDs. Meanwhile, it can be observed that the afterglow intensity from the CDs increases with the enhanced oxidation, implying that the afterglow of CDs originates from the surface oxidation.

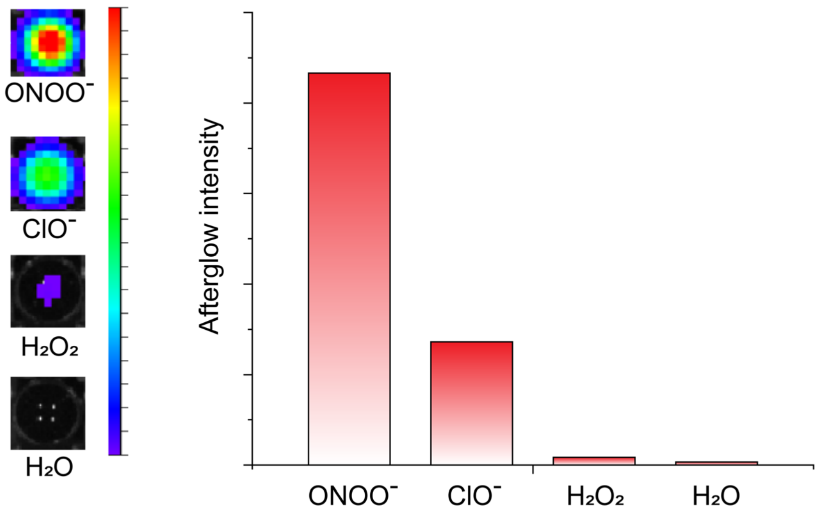

Supplementary Fig. 36. The afterglow intensity of CDs oxidized by common oxidants (active nitrogen, hypochlorous acid, and hydrogen peroxide).

Supplementary Note 9

DFT calculation for the reaction pathways of the polymer unit of CDs and $^1O_2$: Using first-principles calculations based on the density functional theory (DFT), the following [2-2] / retro- [2-

2] reaction process was studied. The configuration optimization and frequency calculation of the above four intermediates and two transition states were carried out, and the free energy broken lines of the above reactions at 298 K were obtained.

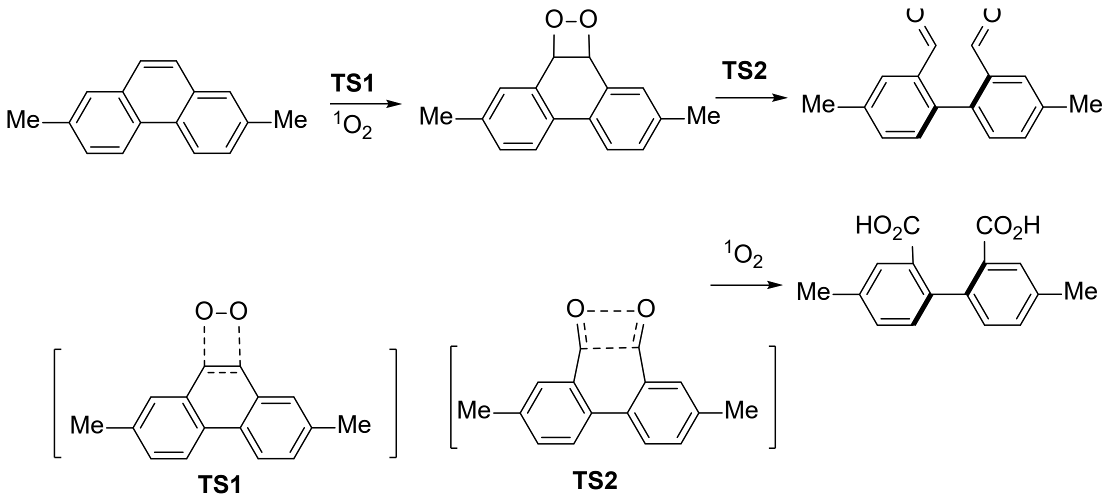

Computational details: The first-principles calculations are based on the density functional theory (DFT) with the projector augmented wave (PAW) method[9] as implemented in the VASP code[10]. The generalized gradient approximation-Perdew-Burke-Ernzerhof (GGA-PBE) method[11] is used to describe the exchange-correlation functions. Dispersion correction of the system is performed using the DFT-D3 method[12] to better describe the vdW interaction. A plane-wave cutoff energy of 400 eV is used. The Brillouin zones were sampled using k-points with $2\pi \times 0.02$ Å^{-1} spacing in the Monkhorst–Pack scheme[13]. All atoms are fully relaxed with a tolerance in total energy of 10^{-5} eV, and the forces on each atom are less than 0.02 eV Å^{-1}. A vacuum of 16 Å perpendiculars to the surface is applied to avoid the interaction between adjacent slabs. The climbing image nudged elastic band (CI-NEB) method[14] is used to determine the energy barriers of the various kinetic processes.

To analysis the size effects of the carbon nanodots (CDs), three simplified models C3, C5, and graphene nanoribbon (GNR) are used for theoretical calculations. The C3 and C5 contain 3 and 5 six-carbon rings (14 and 22 carbon atoms), respectively. The GNR is modeled in armchair shaped with six-unit cells along the periodic direction (48 carbon atoms). The dangling bonds of carbon atoms at edge are saturated by the H atoms.

Supplementary Note 10

We have compared the change in afterglow intensity of the CDs with different sizes over a 20 min period. The initial afterglow intensity is depicted in the Supplementary Fig. 37. As previously mentioned, the initial afterglow intensity of CDs-1 (~GNR) is indeed lower than that of CDs-2 (~C5) and CDs-3 (~C3); however, the afterglow decay rate of CDs-1 (~GNR) is significantly slower than that of CDs-2 (~C5) and CDs-3 (~C3). After 10 min, the afterglow intensity of CNR already becomes stronger than that of CDs-2 (~C5) and CDs-3 (~C3). The consistency between this theory and the explanation regarding CDs afterglow mechanism validates its accuracy.

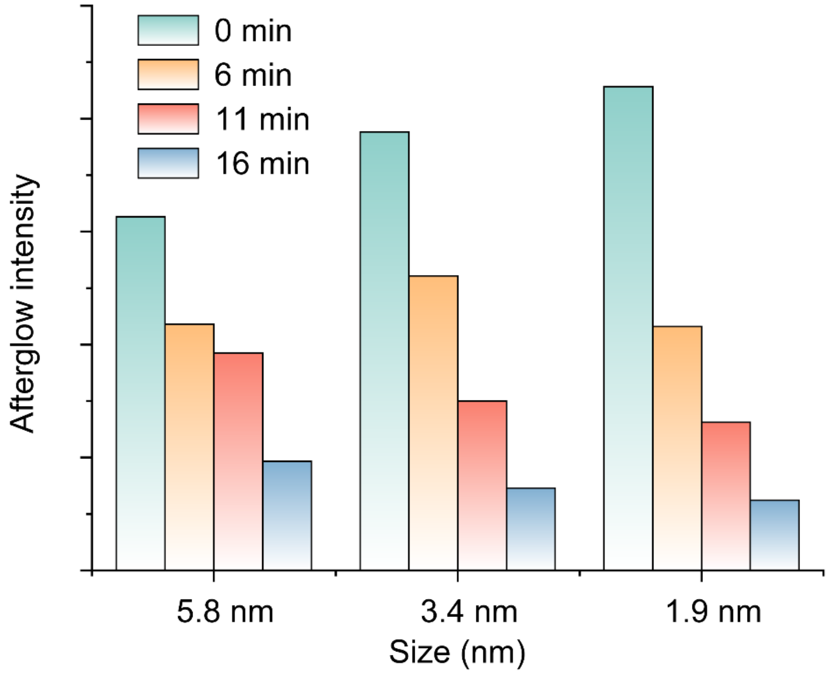

Supplementary Fig. 37. The afterglow decay of CDs-1, CDs-2 and CDs-3 over a 20 min period after irradiation (660 nm, 1.5 W cm^{-2}, 1 mg mL^{-1}).

Supplementary Note 11

The aggregation of p-CDs:

The PL and UV-vis spectra of p-CDs and CDs have been tested. As shown in Supplementary Fig. 38, the p-CDs present the same PL spectra as the initial CDs. Meanwhile, the PL intensity of p-CDs at various concentrations was compared. As illustrated in the Supplementary Fig. 39, with an increase in p-CDs concentration, the PL intensity initially enhances and subsequently diminishes, verifying that there are no Aggregation Induced Emission events in the p-CDs. Meanwhile, the p-CDs prepared with only F127 illustrate nearly no fluorescence (Supplementary Fig. 38a), verifying

that the weak influence of F127 to prepare the afterglow p-CDs. In addition, the UV-Vis absorption spectrum of p-CDs has been also measured. As illustrated in Supplementary Fig. 40, there are no significant difference between the CDs and p-CDs in their absorption spectra, indicating uniform dispersion of CDs in F127 rather than in interparticle aggregation state. This result is also supported by the TEM results from CDs and p-CDs (Supplementary Fig. 41), in which the CDs are evenly and homogeneously dispersed in the F127 polymer.

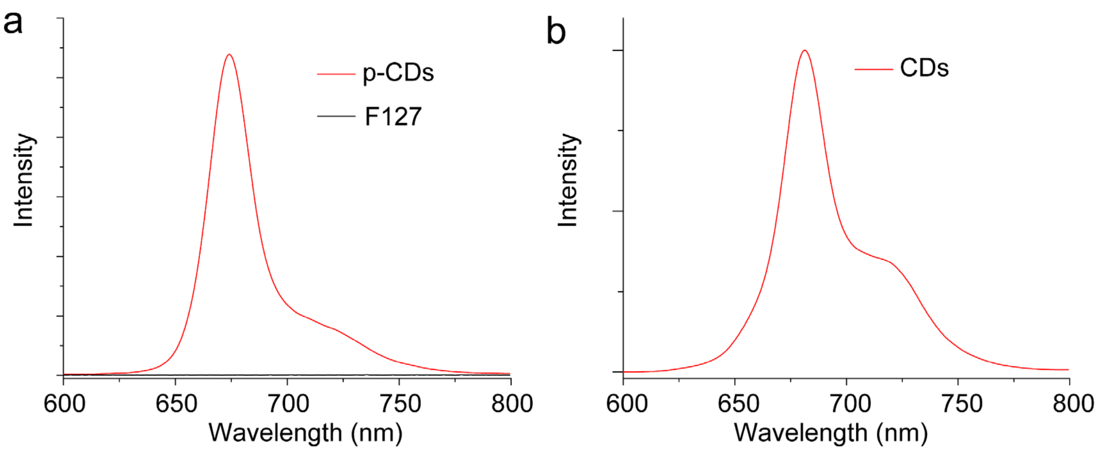

Supplementary Fig. 38. a Fluorescence spectrum of p-CDs and F127, b Fluorescence spectra of CDs.

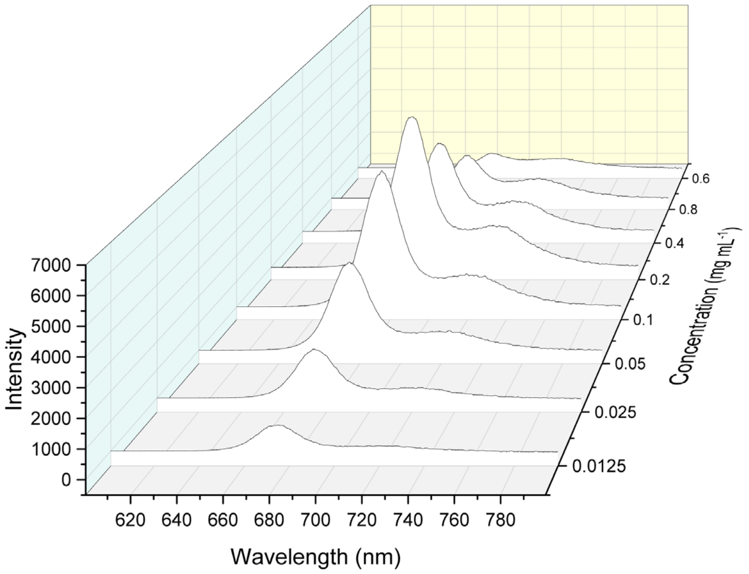

Supplementary Fig. 39. The FL intensity of p-CDs at various concentrations.

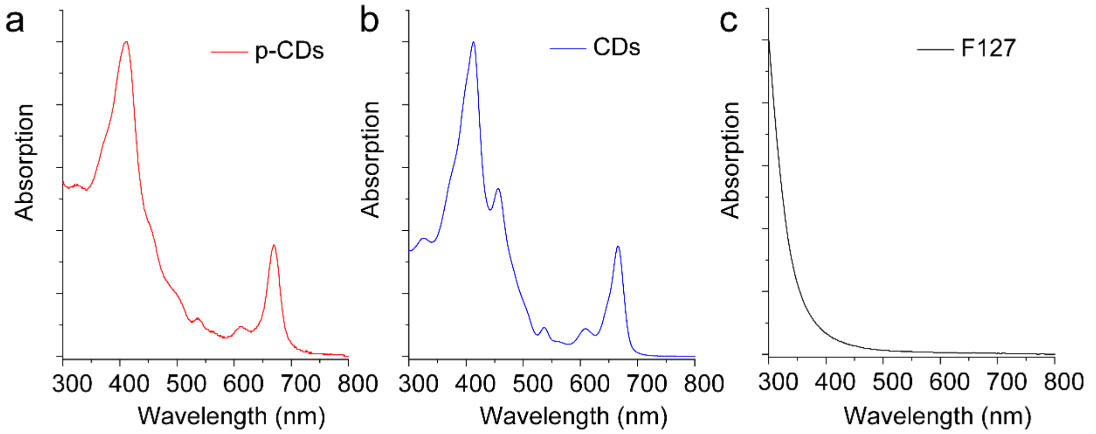

Supplementary Fig. 40. UV-Vis spectra of a p-CDs, b CDs and c F127.

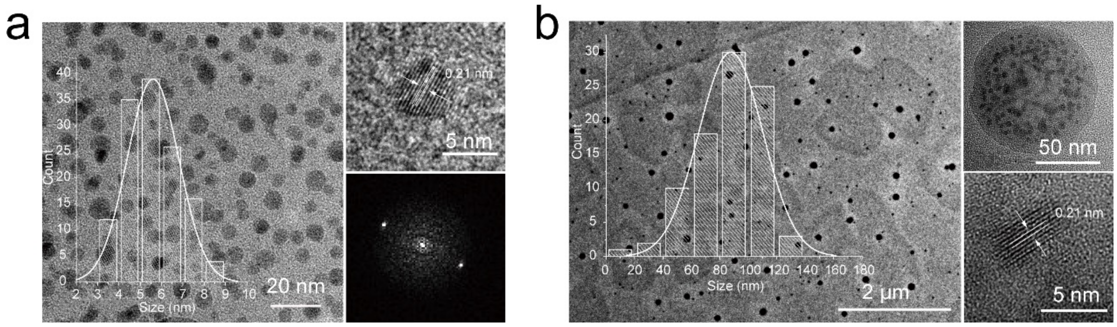

Supplementary Fig. 41. Transmission electron microscopy (TEM, left), high-resolution TEM (HR-TEM, upper right) images and selected area electron diffraction (SAED, lower right) pattern of a CDs and b p-CDs. The imaging in (**a, b**) derived from three independent measurements, and the statistical distribution in (**a,b**) derived from 100 independent measurements.

Supplementary Note 12

We have conducted a $^1O_2$ generation test using p-CDs-1, p-CDs-2, and p-CDs-3 under identical conditions. As shown in Supplementary Fig. 42, there is nearly no significant difference in EPR intensity for the three samples, indicating the similar capability of p-CDs-1, p-CDs-2, and p-CDs-3 to generate $^1O_2$ under the same light irradiation. Thus, the differences in the afterglow lifetimes among p-CDs-1, p-CDs-2, and p-CDs-3 are likely attributed to variations in oxidation rates induced by the size differences of CDs.

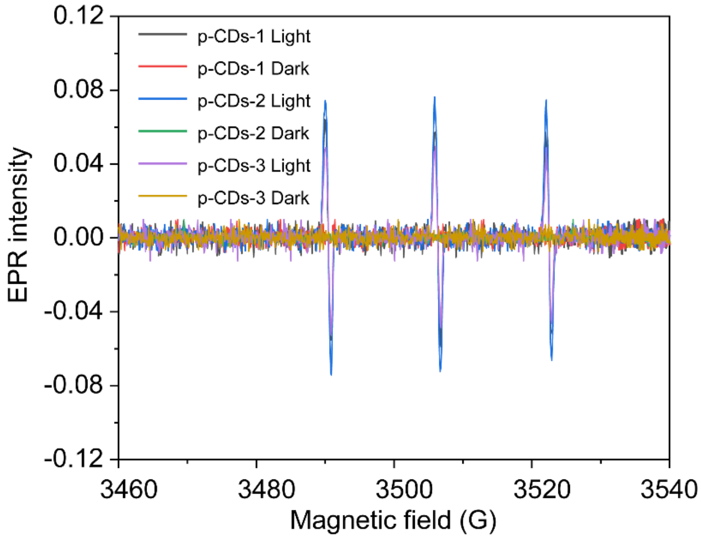

Supplementary Fig. 42. The EPR signals of p-CDs-1, p-CDs-2 and p-CDs-3 under dark and the same light irradiation.

Supplementary Note 13

In this work, the afterglow of the CDs originates from the continuous oxidation of the CDs by singlet oxygen generated from light irradiation. In this process, the oxidation rate of the CDs can determine the afterglow intensity and luminous duration of the CDs. After the CDs is treated by amphiphilic block polymer polyether F127, the CDs changes from fat soluble to hydrophilic. Furthermore, when the CDs are coated by F127 and the surface is passivated, the oxidation rate of the CDs further reduces, and thus the lifetime of p-CDs is greatly improved. Because the oxidation rate of the CDs decreases after coating, the afterglow intensity of p-CDs is obviously weaker than the initial CDs (Supplementary Fig. 43). Actually, it can be found that all the afterglow intensities of the p-CDs with the same size decrease significantly after the coating treatment in comparision with the initial CDs.

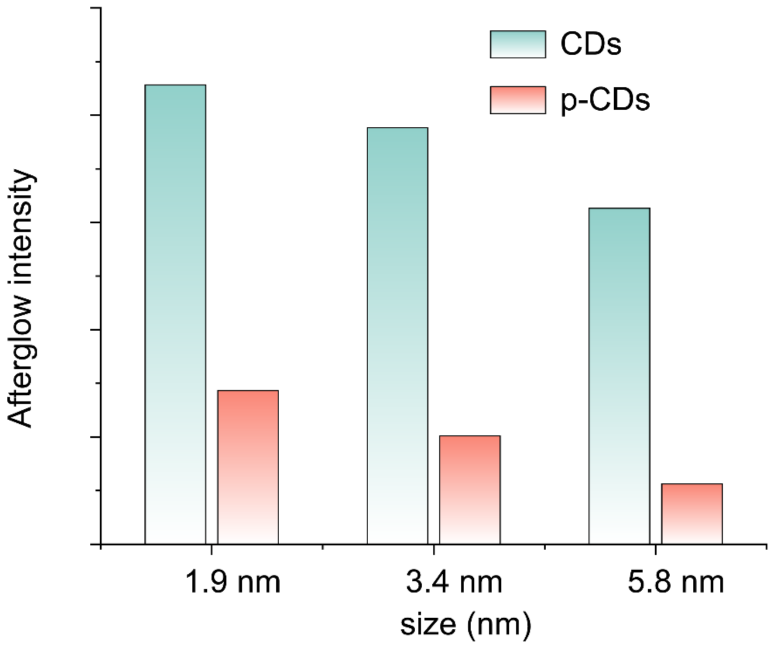

Supplementary Fig. 43. The afterglow intensity of different CDs and p-CDs.

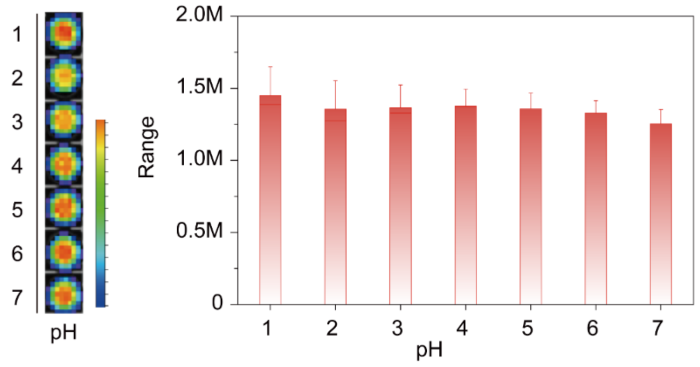

Supplementary Fig. 44. The afterglow photograph and the corresponding afterglow intensity of p-CDs with the pH from 1 to 7 (the concentration of p-CDs: 1 mg mL^{-1}, 660 nm, 1.5 Wcm^{-2}, 2 min).

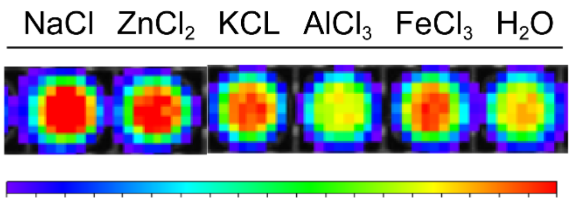

Supplementary Fig. 45. The photo of afterglow of p-CDs with different metal ion (the concentration of p-CDs: 1 mg mL^{-1}, the concentration of metal ion: 1mM, 660 nm, 1.5 W cm^{-2}, 2 min).

Supplementary Note 14

The toxicity of CDs in different tumor cell lines was tested, and the results were illustrated in the Supplementary Fig. 46. At a concentration of 1 mg mL^{-1} p-CDs, the tumor cell lines of MKN-45 exhibit a survival rate of exceeding 90% (Supplementary Fig. 46a), and the tumor cells can maintain the outstanding viability after 2 and 3 days of cultivation (Supplementary Fig. 46b and 46c), indicating the exceptional biocompatibility of p-CDs. Similarly, the p-CDs also present extremely low biological toxicity in the tumor cell lines of KYSE-150 (Supplementary Fig. 46d-46f).

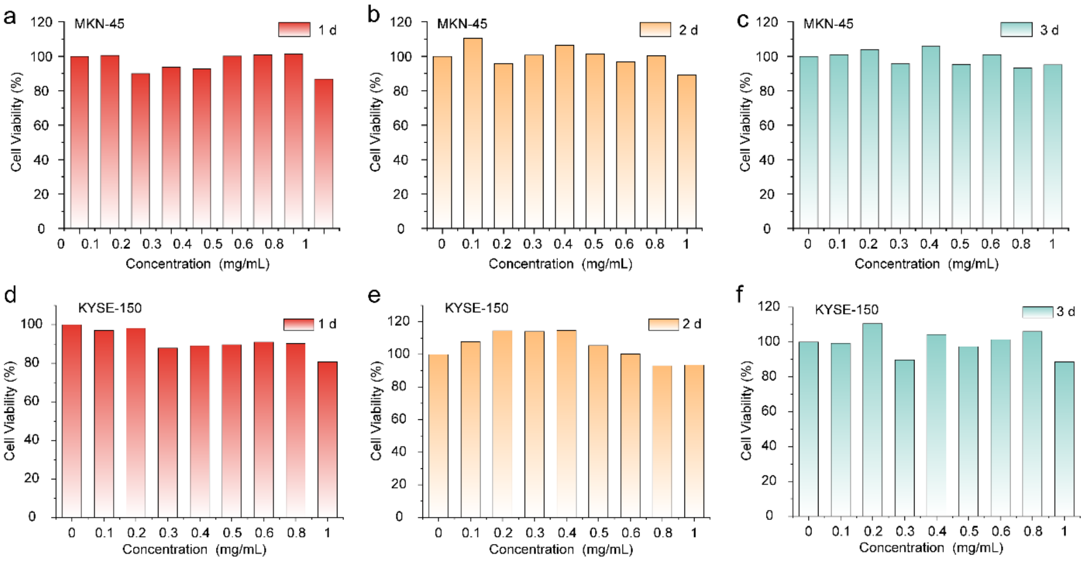

Supplementary Fig. 46. The toxicity of CDs in different tissue cells.

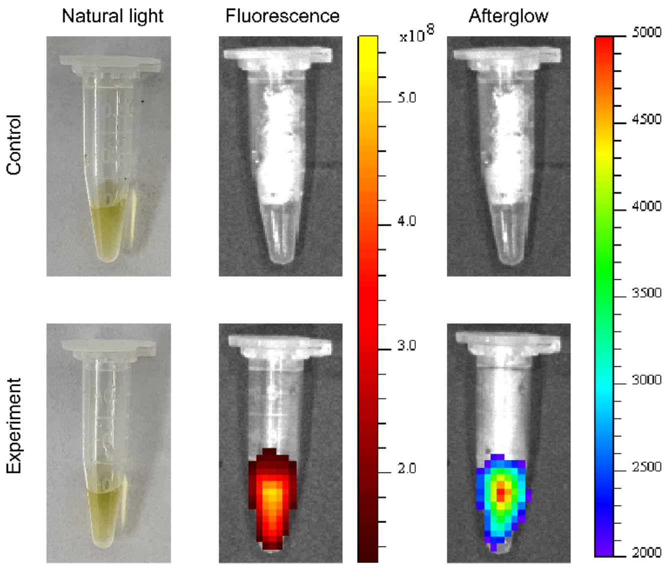

Supplementary Fig. 47. The fluorescence and afterglow of urine of mice after injecting p-CDs solution into the tail vein of mice for 2h.

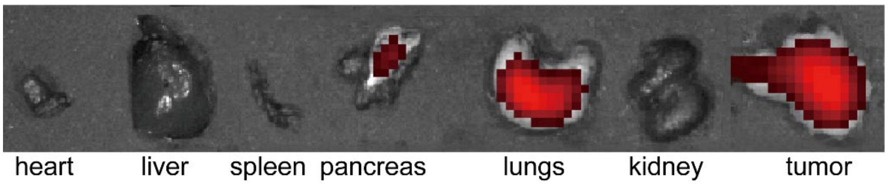

Supplementary Fig. 48. The fluorescence of CDs in organs of mice after injecting p-CDs solution into the tail vein of mice for 24h.

Supplementary Note 15

The purification of CDs:

In the process of solvothermal preparation, different CDs and other molecular fluorophores may be produced. However, the column chromatography purification can efficiently separate the required CDs from the potential formation of 2 different types of CDs or the presence of organic fluorophores (Supplementary Fig. 49). We believe the column chromatography is one valid technology to obtain pure CDs as reported in previous literatures[15].

The detail of the preparation of CDs and the column chromatography purification: Firstly, the source/supplier of the plant (Solanum nigrum L) was purchased from Bozhou Kangyiyin Biotechnology Co., Ltd. to prepare the afterglow CDs without any additional treatment. In detail, 1 g plant sources were dispersed into 20 mL ethanol and the mixture were transferred to a poly (tetrafluoroethylene) Teflon-lined autoclave (50 mL) and heated for 4 h at 80, 120 and 160 °C, respectively. After the products cooled down to room temperature, the solutions were filtered through a 0.22 μm polyether sulfone membrane to remove large particles, and the obtained crude products were further purified via a silica column chromatography with the eluent of ethyl acetate and petroleum ether (V_{EA} : V_{PE} = 3:1). After removing the solvent under reduced pressure, the CDs-1, CDs-2 and CDs-3 were collected for further characterization.

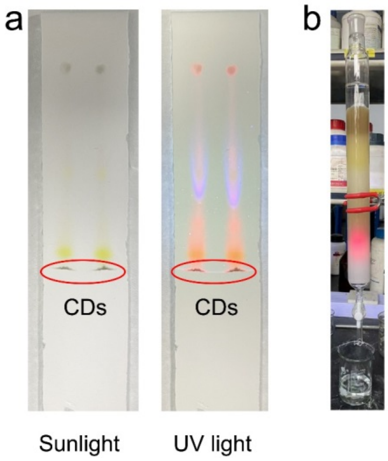

Supplementary Fig. 49. a The photograph of the silica gel plate with the CDs in the eluent for the column chromatography. **b** The photograph of the purification process (The red emission is produced by irradiation at 365 nm).

Supplementary Table 1. The lifetime fitted results of p-CDs-1, p-CDs-2 and p-CDs-3.

**Sample**	**a_1**	**t_1 (min)**	**a_2**	**t_2 (min)**	**T_{ave} (min)**	**T_{ave} (h)**
**p-CDs-1**	2.39703	2.93	199816.2	383.05	351.10168	5.85169
**p-CDs-2**	1.3741E6	4.96	254494.6	276.63	252.65087	4.21085
**p-CDs-3**	697145.5	7.08	293251.8	218.81	203.68668	3.39478

Supplementary Table 2. Comparison of the lifetime of long afterglow materials.

	Materials	λem (nm)	Lifetime	Ref.
CDs	p–n-CD	778 nm	185 ms	17
	m-CDs@CA	425 nm	1.26 s	18
	B-CDs	476-532 nm	2.61 s	19
	CDs@MnAPO-CJ50	620 nm	10.94 ms	20
	CDs	462 nm	2.03 s	21
	CNDs@SiO_2	520 nm	1.86 s	22
	CDs@SiO_2	464 nm	5.72 s	23
	CDs	600 nm	109.6 ms	24
	CNDs@SiO_2@ER	510 nm	1.44 s	25
Organic	$MeOTPP^+$/mCBP	624 nm	610 s	26
	TMB/PPT	526 nm	2.20 s	27
	DPhCzT	575 nm	1.35 s	28
	NCBS/DP	780 nm	110 s	29
	NCBS/DP	780 nm	4.8 min	30
	SPN-NCBS	780 nm	396 s	31
	$F1^{2+}$-ANP -Gal	580 nm	6.6 min	32
Inorganic	$ZnGa_{1.995}Cr_{0.005}O_4$	695 nm	50 s	33
	$ZnGa_2O_4Cr_{0.004}$	696 nm	200 s	34
	$ZnGaO_4$:Cr-IR780	697 nm	6 min	35
	$ZnGa_2O_4$:Cr^{3+}	690 nm	100 s	36
	2D Perovskites	490 nm	480 ms	37
	AlN	352 nm	9360 s	38

References

1. Chen, R. *et al.* Ultra-narrow-bandwidth deep-red electroluminescence based on green plant-derived carbon dots. *Adv. Mater.* **35**, 2302275, (2023).
2. Chen, W. C. *et al.* Achieving efficient violet-blue electroluminescence with CIEy <0.06 and EQE >6% from naphthyl-linked phenanthroimidazole–carbazole hybrid fluorophores. *Chem. Sci.* **8**, 3599–3608, (2017).
3. Lou, Q. *et al.* Carbon nanodots with nearly unity fluorescent efficiency realized via localized excitons. *Adv. Sci.* **9**, 2203622, (2022).
4. Liu, J. *et al.* Deep Red Emissive Carbonized polymer dots with unprecedented narrow full

width at half maximum. *Adv. Mater.* **32**, 1906641, (2020).

5. Liu, J. *et al.* One-step hydrothermal synthesis of nitrogen-doped conjugated carbonized polymer dots with 31% efficient red emission for in vivo imaging. *Small* **14**, 1703919, (2018).
6. Dong, S. *et al.* Rapid screening of oxygen states in carbon quantum dots by chemiluminescence probe. *Anal. Chem.* **89**, 12520–12526, (2017).
7. Zhou, W. *et al.* Insights into the role of nanostructure in the sensing properties of carbon nanodots for improved sensitivity to reactive oxygen species in living cells. *Chem. Commun.* **53**, 2122–2125, (2017).
8. Zhao, L. et al. Chemiluminescence of carbon dots under strong alkaline solutions: a novel insight into carbon dot optical properties. *Nanoscale* **5**, 2655–2658, (2013).
9. Blochl, P. E. Projector augmented-wave method. *Phys. Rev. B* **50**, 17953–17979 (1994).
10. Kresse, G., Furthmüller, J. Efficient iterative schemes for ab initio total-energy calculations using a plane-wave basis set. *Phys. Rev. B* **54**, 11169 (1996).
11. Perdew, J. P., Burke, K. and Ernzerhof, M. Generalized gradient approximation made simple. *Phys. Rev. Lett.* **77**, 3865–3868 (1996).
12. Grimme, S., Antony, J., Ehrlich, S. and Krieg, S. A consistent and accurate ab initio parametrization of density functional dispersion correction (DFT-D) for the 94 elements H-Pu. *J. Chem. Phys.* **132**, 154104 (2010).
13. Monkhorst, H. J., Pack, J. D. Special points for Brillouin-zone integrations. *Phys. Rev. B* **13**, 5188 (1976).
14. Henkelman, G., Uberuaga, B. P., and Jonsson, H. A climbing image nudged elastic band method for finding saddle points and minimum energy paths. *J. Chem. Phys.* **113**, 9901 (2000).
15. Yuan, F. *et al.* Bright high-colour-purity deep-blue carbon dot light-emitting diodes via efficient edge amination. *Nat. Photonics* **14**, 171–176, (2020).
16. Liu, J. *et al.* Mulberry-leaves-derived red-emissive carbon dots for feeding silkworms to produce brightly fluorescent silk. *Adv. Mater.* 3**4**, 2200152, (2022).
17. Geng, B. *et al.* Near-infrared phosphorescent carbon dots for sonodynamic precision tumor therapy. *Nat. Commun.* **13**, 5735, (2022).
18. Jiang, K. *et al.* Enabling robust and hour-level organic long persistent luminescence from carbon dots by covalent fixation. *Light Sci. Appl.* **11**, 80, (2022).

19. Feng, Q., Xie, Z., Zheng, M. Colour-tunable ultralong-lifetime room temperature phosphorescence with external heavy-atom effect in boron-doped carbon dots. *Chem. Eng. J.* **420,** 127647, (2021).
20. Wang, B. *et al.* Carbon dots in a matrix: Energy-transfer-enhanced room-temperature red phosphorescence. *Angew. Chem. Int. Ed.* **58**, 18443–18448, (2019).
21. Song, Z. *et al.* A molecular engineering strategy for achieving blue phosphorescent carbon dots with qutstanding efficiency above 50%. *Adv. Mater.* **35**, 2207970, (2022).
22. Liang, Y. *et al*. Ultralong and efficient phosphorescence from silica confined carbon nanodots in aqueous solution. *Nano Today* **34**, 100900, (2020).
23. Sun, Y.; *et al*. Ultralong lifetime and efficient room temperature phosphorescent carbon dots through multi-confinement structure design. *Nat. Commun.* **11**, 5591, (2020).
24. Lou, Q. *et al*. Thermally enhanced and long lifetime red TADF carbon dots via multi-confinement and phosphorescence assisted energy transfer. *Adv. Mater.* **35**, 2211858, (2023).
25. Lv, W. *et al*. Enhanced phosphorescence of carbon nanodots via double confinement for 3D artworks with long emission lifetimes. *Small* **19**, 2302504, (2023).
26. Jinnai, K., Kabe, R., Lin, Z., Adachi, C. Organic long-persistent luminescence stimulated by visible light in p-type systems based on organic photoredox catalyst dopants. *Nat. Mater.* **21**, 338–344, (2022).
27. Kabe, R., Adachi, C. Organic long persistent luminescence. *Nature* **550**, 384–387, (2017).
28. An, Z. *et al*. Stabilizing triplet excited states for ultralong organic phosphorescence. *Nat. Mater.* **14**, 685-90, (2015).
29. Xu, C., Huang, J., Jiang, Y., He, S., Zhang, C., Pu, K. Nanoparticles with ultrasound-induced afterglow luminescence for tumour-specific theranostics. *Nat. Biomed. Eng.* **7**, 298–312, (2023).
30. Xie, C., Zhen, X., Miao, Q., Lyu, Y., Pu, K. Self-assembled semiconducting polymer nanoparticles for ultrasensitive near-infrared afterglow imaging of metastatic tumors. *Adv. Mater.* **30**, 1801331, (2018).
31. Miao, Q. *et al*. Molecular afterglow imaging with bright, biodegradable polymer nanoparticles. *Nat. Biotechnol.* **35**, 1102–1110, (2017).
32. Wu, L. *et al.* H_2S-activatable near-infrared afterglow luminescent probes for sensitive

molecular imaging in vivo. *Nat. Commun.* **11**, 446, (2020).

33. Maldiney, T. *et al*. The in vivo activation of persistent nanophosphors for optical imaging of vascularization, tumours and grafted cells. *Nat. Mater.* **13**, 418–426, (2014).

34. Li, Z. *et al*. Direct aqueous-phase synthesis of sub-10 nm "luminous pearls" with enhanced in vivo renewable near-infrared persistent luminescence. *J. Am. Chem. Soc.* **137**, 5304–5307, (2015).

35. Liu, G. *et al*. "Wax-sealed" theranostic nanoplatform for enhanced afterglow imaging-guided photothermally triggered photodynamic therapy. *Adv. Funct. Mater.* **28**, 1804317, (2018).

36. Chen, Z. *et al*. Low dose of X-ray-excited long-lasting luminescent concave nanocubes in highly passive targeting deep-seated hepatic tumors. *Adv. Mater.* **31**, 1905087, (2019).

37. Ge, L. *et al*. Stimulating efficient and stable ultralong phosphorescence of 2D perovskites by dual-mode triplet exciton stabilization. *Chem. Mater.* **34**, 8917–8924, (2022).

38. Lin, R *et al*. X-ray radiation excited ultralong (>20,000 seconds) intrinsic phosphorescence in aluminum nitride single-crystal scintillators. *Nat. Commun.* **11**, 4351, (2020).
