# Supplementary material for: Photooxidation triggered ultralong afterglow in carbon nanodots
Source: Nat Commun. 2024 Mar 15;15:2365. doi: 10.1038/s41467-024-46668-z (PMC10943204; doi:10.1038/s41467-024-46668-z)
Supplement: Supplementary file 3 — Description of Additional Supplementary Files [file 41467_2024_46668_MOESM3_ESM.pdf]

**Description of Additional Supplementary Files**

**File Name: Supplementary Data 1**
**Description:** The atomic coordinates of models (C3, C5 and GNR).
